# Pharmacists’ clinical knowledge and practice in the safe use of contraceptives: real knowledge vs. self-perception and the implications

**DOI:** 10.1186/s12909-021-02864-9

**Published:** 2021-08-16

**Authors:** Ana Golić Jelić, Ljiljana Tasić, Ranko Škrbić, Valentina Marinković, Svjetlana Stoisavljević Šatara, Nataša Stojaković, Vanda Marković Peković, Brian Godman

**Affiliations:** 1 Department of Pharmacology, Toxicology and Clinical Pharmacology, University of Banja Luka – Medical Faculty, Save Mrkalja 14, 78000 Banja Luka, Bosnia and Herzegovina; 2 Faculty of Pharmacy, Department of Social pharmacy and Pharmaceutical legislation, University of Belgrade, Vojvode Stepe 450, 11221 Belgrade, Serbia; 3 Medical Faculty, Department of Social Pharmacy, University of Banja Luka, Save Mrkalja 14, Banja Luka, Bosnia and Herzegovina; 4 Institute of Pharmacy and Biomedical Sciences, University of Strathclyde, Scotland Glasgow, UK; 5 School of Pharmacy, Sefako Makgatho Health Sciences University, Ga-Rankuwa, Pretoria, South Africa

**Keywords:** Pharmacist, Clinical knowledge, Self-assessment, Case scenario, Oral and emergency contraceptives, Preceptor, Bosnia and Herzegovina

## Abstract

**Background:**

Pharmacists are often the first healthcare professionals that patients contact with their illnesses and requests for medical information, which is enhanced following the recent COVID-19 pandemic. Community pharmacists are expected and required to possess a broad spectrum of knowledge and skills. Self-assessment of these competencies is needed for their self-improvement.

**Purpose of the study:**

To assess pharmacists’ clinical knowledge and practice in the safe use of contraceptives, and to compare the scores obtained by external observation with pharmacists’ self-assessment of their knowledge as well as investigate the significance of preceptorship experiences. Contraceptives was chosen as the subject area in view of high rates of abortions as a means of contraception in Bosnia and Herzegovina.

**Methods:**

A questionnaire approach was used. The questionnaire included the following: the first domain contained two case scenarios (safe use of contraceptives), which evaluated clinical knowledge, a second domain in which pharmacists self-assessed their knowledge to resolve cases from the first domain and a third domain that measured the demographics of pharmacists (including experience in preceptorship). Dispensing practice was evaluated in the second domain. The questionnaires were distributed to a convenient sample of 100 pharmacists at the Annual Meeting of Bosnia and Herzegovina Pharmacists. The results were presented as counts (%). The groups (preceptors and non-preceptors) were compared using Mann-Whitney U test, paired assessments were analyzed by Wilcoxon signed-rank test and Spearman’s correlation was used to assess the correlation between variables.

**Results:**

Of the 100 pharmacists invited to participate, 84 completed the questionnaire (84 % response rate). There was no agreement between pharmacists’ real knowledge (average score - *case 1*: 2.71, *case 2*: 3.3) and their self-assessment (average score - *case 1*: 3.77, *case 2*: 3.91). There was no statistically significant difference in the actual knowledge of pharmacists (experienced/non-experienced in precepting), while the difference in the self-assessment was significant between these two groups.

**Conclusion:**

Pharmacists appear to overrate themselves, which leads to self-enhancement bias, in which the experience in precepting has some influence. Pharmacists’ capability in performing an objective self-assessment of their clinical knowledge needs to be carefully studied in the future to fully benefit patients.

**Supplementary Information:**

The online version contains supplementary material available at 10.1186/s12909-021-02864-9.

## Background

Community pharmacists’ roles are evolving, and enhanced following the recent COVID-19 pandemic [1–3]. This builds on their role as typically the first healthcare professionals that patients contact for a range of illnesses [4]. Currently, community pharmacists are expected to deal with a range of medicine-related problems, improve patients’ adherence to medicines especially those with chronic asymptomatic diseases, help enhance patients’ quality of life, deal with pharmacoeconomic and affordability issues as well as give appropriate and acceptable solutions and advice [5, 6]. The urgency of patients to solve their problems as soon as possible in an acceptable manner makes community pharmacies often the first place they visit, enhanced by long waiting times to see physicians in ambulatory care in a number of countries and lack of resources to fund both medicines as well as physician costs [4, 7]. Affordability is a particular concern in a number of low- and middle-income countries (LMICs) where long-term illness can have a serious consequence on families [8, 9].

Consequently, working in a community pharmacy, which requires a mix of competencies that may well overlap with other disciplines, and knowledge in order to handle the demands, is an increasing challenge for pharmacists today [4]. The required knowledge and skills of community pharmacists now includes good communication skills; knowledge of drug therapy, nondrug therapy and complementary medicine; knowledge of diseases; laboratory and diagnostic skills; physical assessment skills; therapeutic planning skills and critical evaluation of drug information skills [10, 11]. Knowledge of drug therapy and disease management has been facilitated in the Republic of Srpska and other countries by the production of easy-to-use guidelines of common illnesses encountered in ambulatory care [12]. Critical evaluation skills have been emphasized in the recent COVID-19 pandemic given the level of misinformation especially surrounding possible treatments such hydroxychloroquine with the potential to increase deaths from suicide as well as appreciably increase prices through shortages [13–15]. All these competencies represent key challenges facing healthcare professionals including community pharmacists today [16]. We are aware that the discipline of pharmaceutical care arose from dissatisfaction with previous practice norms and the pressing need for more competent healthcare professionals with comprehensive knowledge of the therapeutic use of medicines to improve the care of patients [17], and this will continue.

This improved knowledge and skills can enhance patient care as seen by pharmacists efficiently improving the care and management of patients with COPD in India [18, 19], with similar findings in other middle-income countries [20, 21]. Community pharmacists are also heavily involved with guiding patients on appropriate management of their acute respiratory tract infections through issuing guidelines and training to pharmacists[12], and have a key opportunity to be leaders of antibiotic stewardship across countries especially where there is still self-purchasing of antibiotics despite legal concerns [22, 23].

Different assessments tools are used to assess pharmacists’ professionalism and competencies [24, 25]. Case scenario (including clinical cases with several multiple-choice statements) is a good evaluation tool, especially in aspects of patient safety [26].

Self-assessment is considered an important skill for development, and within professional practice, self-assessment is the foundation upon which the cycle of continuing professional development is built [27, 28]. Through self-assessment, health professionals develop the ability to manage their self-improvement [16, 29]. Whilst self-assessment is used to describe many types of activities, aspects of “self-rating” or “self-audit” used to assess knowledge or clinical performance would appear to be the most relevant to pharmacists [30]. Overall, we believe the quality of pharmacy education will be improved with requirements for direct measures of students’ learning beyond their knowledge and skills [31]. However, we are currently unaware of published research on the self-assessment skills that pharmacists need to possess to improve patient care, which is a concern as healthcare professionals can overrate their performance [32]. This is different than the situation with physicians where some published research has shown limited ability for accurately self-assessment [30, 33]. In addition, gaps in pharmacists’ expertise can lead them to make mistakes and errors, and fail to recognize when they are acting inappropriately [34]. Having said this, health professionals who are not that confident may be preferable because they may well check unknown facts before acting whereas overconfident professionals may not [33].

We were particularly interested in pharmacists knowledge regarding contraception in Bosnia and Herzegovina (B & H) since it is estimated worldwide 3.9 million girls aged 15 to 19 undergo unsafe abortions each year [35]. In addition, in B & H 28.8 % of women in a survey undertaken in 2010 had an induced abortion with an abortion the preferred method of birth control among married woman (88.6 %) and girls in secondary school (64.5 %) [36]. This is a potential concern in view of a negative impact on mental health, and consequently needs to be addressed [36]. This also applies to other Balkan countries where there can be an abortion culture [37]. For instance, the abortion rate in Serbia is currently double the fertility rate, and among the highest rates in Europe [38]. Alongside this, the graduate curricula for pharmacists in the Republic of Srpska at this point of time does not include pharmacy practice tutorials and practice counselling in the simulation laboratory. However, there is mandatory graduate practice in the pharmacy in last semester where all the acquired knowledge is practiced to some extent. Within the Organization of the Pharmaceutical Activity course, students are taught about pharmaceutical care, which includes pharmacists providing a patient-centered approach to health care. In addition, the course of Pharmacology with Pharmacotherapy includes a clinical approach, which includes issues about hormonal therapy and oral and emergency contraceptives. Pharmacy students who started their studies with the new curricula, from 2019, will have lectures and practice with a focus on incorporating a patient-centered approach, e.g., Objective Structured Clinical Examination – OSCE scenarios, in the last (fifth) year. After graduation, each pharmacist needs to complete a year long internship in addition to the requirement to take examinations at the Ministry of Health and Social Welfare and at the Pharmaceutical Chamber in order to become a registered pharmacist capable of working independently [39]. This year-long internship includes 300 days in a community pharmacy, 30 days at a control drug laboratory and 35 days in a hospital pharmacy. Each intern pharmacist needs to have a preceptor (registered pharmacist with experience in teaching interns) in each of the institutions during their internship. A similar situation with curricula and internship exists in Federation of Bosnia and Herzegovina. We are aware that pharmacy provision allows for a more direct access to potential methods to help with sexual and reproductive health (SRH), in which contraceptives play an important part [40]. In addition, we believe lowering barriers to SRH approaches does not necessarily increase sexually risky behavior [40]. Currently in B & H, oral and emergency contraceptives are the most dispensed medical contraception in pharmacies, while intrauterine devices and long-acting injections used in hospitals [41]. Mifepristone/misoprostol, vaginal rings and implants are currently not registered in B & H [41]. Currently, pharmacists in B & H are only allowed to dispense ulipristal acetate for emergency contraception, as an over-the-counter (OTC) medicine, without a physicians’ prescription. Having said this, there is no regulatory requirement for declining to dispense ulipristal acetate to any female regarding their age, including adolescents [41]. However, in view of identified concerns in B & H we are aware that pharmacy provision in SRH requires competent pharmacists, which is not possible without being equipped with the necessary knowledge and skills [42, 43]. Consequently, the aims of the study were firstly to assess pharmacists’ clinical knowledge and practice regarding the safe use of contraceptives; secondly, to compare the scores obtained by external observation with pharmacists’ self-assessment of their knowledge, and thirdly to investigate the significance of preceptorship experiences in enhancing professional competency. The findings can be used to provide guidance not only to improve family planning in B & H and beyond but also add to knowledge regarding self-assessment by pharmacists given limited published knowledge in this area. For the purposes of our research, self-assessment will be defined in terms of the accuracy of predicting one’s own perception of knowledge compared with an objective standard.

## Methods

### Study design

A cross-sectional survey of pharmacists was conducted in B & H, which consists of the two entities, the Republic of Srpska and the Federation of B & H. Each entity is responsible for the healthcare and education on its territory, as well as the Brcko District of B & H. The survey was conducted on the 21st February 2020. The two cases with accompanying questions were developed between December 2019 to February 2020. The questionnaire was designed to address the aims of the study and used a qualitative method, the nominal group technique (NGT). The NGT is a highly structured face-to-face group interaction method that empowers participants by providing an opportunity to have their voices heard and opinions considered by others, and is often used in research projects [44–48]. We conducted three NGT sessions (two homogenous and one heterogeneous). The first homogenous session was conducted with four clinical pharmacologists, and the second session with three experienced pharmacy practice researchers. A heterogeneous NGT session (two pharmacists, one psychologist that is also a pharmacist, and two gynecologists) was subsequently conducted with the aim of validating the results from the first sessions. These small numbers of NGT participants allows for maximal and in-depth contributions from all members; furthermore, these three round sessions were deemed adequate since no new themes emerged by the end of the third round which is similar to other studies [49, 50]. The NGT sessions were conducted in January 2020 at the University of Banja Luka and at the University of Belgrade. Each group session lasted for two hours. All NGT sessions comprised four key stages: silent generation, a round robin, clarification and voting (ranking or rating), with a draft questionnaire sent beforehand based on available literature [51]. The sums of the ranks from homogenous sessions were used to classify and clarify the ideas that were the most important to the members of two NGTs. After finalizing the questionnaire according to participants’ opinions, we subsequently e-mailed the questionnaire to the members of the heterogeneous NGT session. Their role in the session was to add any ideas they thought had been overlooked and to rank (in their opinion) the statement ideas for the first domain of the questionnaire for each *case scenario* (from 1-least important to 10-most important). Afterwards, the questionnaire was independently pretested regarding the clarity, preciseness and intelligibility of the questions by five pharmacists not associated with the research team and selected via the snowball methodology [52]. After completing the questionnaire, each pharmacist had a brief interview with the principal investigator to discuss the findings. Final adjustments were subsequently made to the questionnaire based on the pretesting pharmacists’ comments. The final version consisted of three domains (Supplementary Material). The first domain was divided into two presented *case scenarios* from practice. *Case 1* was created to assess pharmacists’ knowledge and attitudes in recognizing the symptoms of possible adverse drug reactions of oral contraceptives. *Case 2* was created in order to assess whether emergency contraceptive pills (ECPs) were appropriate and dispensed with suitable counselling advice. In both cases, we wanted to assess whether appropriate advice on the safe use of oral and emergency contraceptives was provided to the patients. Further details of the two scenarios are given in Table 1. Pharmacists had to choose the one answer they thought was correct for each claim (9–10 claims with 2 to 3 possible answers). The last paragraph in each case was open with pharmacists having the option of writing additional advice for the patient. Knowledge about each *case* was assessed through the sum of scores for each claim (the score for each claim was defined in the NGT sessions, according to clinical significance of each). The second domain was designed in order to gather information about pharmacists’ self-evaluation of their knowledge from the presented cases (from the first domain) and about their daily dispensing practice of oral and emergency contraceptives. The third domain was used to collect the demographic data and information on the working experience of the pharmacists.

**Table 1:** Patient case scenarios

Case scenario 1	Case scenario 2
26-year-old woman	16-year-old girl
Planning a trip to the USA in two days	Had unprotected sex 4 nights ago
Unexplained swelling and severe pain in calf of one leg	Long-term boyfriend; interrupt intercourse as contraception
Morning headache	Never went to gynecologist
No leg trauma, no chronic conditions	Religious family
Smoker	Smoker
Taking ^a^COC regularly	

^a^*COC* combined oral contraceptive

Pharmacists’ knowledge (score) and self-evaluation for each *case scenario* was defined as follows: *unsatisfactory, not good enough, average, good* and *very good*. Scores for pharmacists’ knowledge were obtained through external evaluation by the main investigator (A.G.J.) according to the scoring system established in the NGT sessions. The scoring system is available on request from corresponding author (A.G.J).

### Sampling and data collection

The questionnaires were distributed to a convenience sample of 100 out of 1090 pharmacists registered with the Pharmaceutical Chamber of the Republic of Srpska. The pharmacists completed the questionnaires during the Annual Meeting of Bosnia and Herzegovina Pharmacists. All the questionnaires (100) were distributed by the investigators at the same time in the same conference hall, and pharmacists had 45 min time for completion. Pharmacists were asked to complete the questionnaires by themselves without conferring with others. One hundred pharmacists were deemed acceptable since no research had been undertaken in this area to guide sample sizes.

### Statistical analysis

The results are presented as counts (%). The groups (preceptors and non-preceptors) were compared using nonparametric test (Mann-Whitney U test) while paired assessments were analyzed using the Wilcoxon signed-rank test. To assess the correlation between variables, Spearman’s correlation was used. All p-values less than 0.05 were considered significant. All data were analyzed using SPSS 20.0 (IBM Corp. Released 2011. IBM SPSS Statistics for Windows, Version 20.0. Armonk, NY: IBM Corp).

## Results

Out of 100 pharmacists, 84 responded to the questionnaire, which resulted in a response rate of 84 %.

### Demographics of pharmacists and their dispensing practice

More than 90 % of the respondents were female, and most of the respondents were in the age range of 25–35 years. Most of the pharmacists were community pharmacists with preceptor and managerial experience but no previous working experience outside of community pharmacy (Table 2). Of the 84 pharmacists who responded, nearly 90 % dispensed two or more oral contraceptive prescriptions monthly. This compares with 41 % who dispensed two or more emergency contraceptives in a month. 85 % of pharmacists would decline to provide ECP as the female patient in the case scenario was under 18 years old ( e.g. 16 years old).

**Table 2:** Demographics of pharmacists and their dispensing practice

	Pharmacists (*n* = 84)
Age	
<25	0
25–35	54 (65.1 %)
36–45	21 (25.3 %)
46–55	5 (6.0 %)
>56	3 (3.6 %)
Gender female	76 (95.0 %)
Average dispensing number of oral contraceptives per month	
0–1	8 (10.3 %)
2–10	46 (59.0 %)
>10	24 (30.8 %)
Average dispensing number of emergency contraceptives per month	
0–1	46 (59.0 %)
2–10	30 (38.5 %)
>10	2 (2.3 %)
Work in	
community pharmacy	75 (96.2)
hospital pharmacy	1 (1.3)
other	2 (2.6)
Years of experience	
1–5	35 (43.2)
6–10	30 (37)
11–20	10 (12.3)
>20	6 (7.4)
Previous experience	
pharmacy	59 (77.6)
marketing	4 (5.3)
production	1 (1.3)
other	12 (15.8)
Experience as a manager	44 (54.3)
Preceptorship (Mentorship)	49 (61.3)
Preceptor experience	
<5	36 (75)
6–10	8 (16.7)
11–20	3 (6.3)
>20	1 (2.1)

Results are presented as count (%)

### Pharmacists’ real knowledge of the presented case scenario

For case 1, 69 % of pharmacists had a low score (unsatisfactory, not good enough and average) regarding knowledge about oral contraceptives. However, 56 % of pharmacists had good or very good knowledge about the safe use of emergency contraceptives (case 2). See Table 3.

**Table 3:** Pharmacists’ knowledge (real score)

	Pharmacists (*n* = 84)
Case scenario 1	
Unsatisfactory	20 (23.8 %)
Not good enough	23 (27.4 %)
Average	15 (17.9 %)
Good	13 (15.5 %)
Very good	13 (15.5 %)
Case scenario 2	
Unsatisfactory	29 (34.5 %)
Not good enough	7 (8.3 %)
Average	1 (1.2 %)
Good	3 (3.6 %)
Very good	44 (52.4 %)

Results are presented as count (%)

### Agreement between real knowledge and self-assessment of pharmacists

**Fig. 1:**
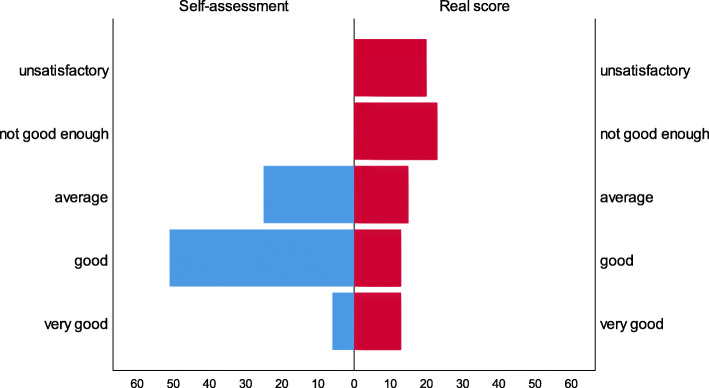
Self-assessment of pharmacists’ knowledge vs. real score knowledge of Case 1

There was no agreement between the knowledge score and self-assessment in *case 1* (Spearman’s Rho=-0.066, *p *= 0.557), although the difference was statistically significant (Wilcoxon Signed Ranks test Z=-5.326, *p* < 0.001). All scores are higher in the self-assessment (Fig. 1).

**Fig. 2:**
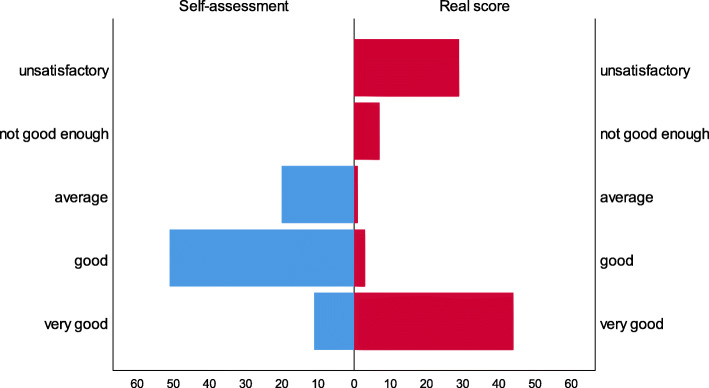
Self-assessment of pharmacists’ knowledge vs. real score knowledge of Case 2

There was though a degree of agreement between knowledge scores and self-assessment in *case 2* (Spearman’s Rho = 0.317, *p* = 0.004), with a statistically significant difference (Wilcoxon Signed Ranks test Z=-3.857, *p* < 0.001). All scores were higher in the self-assessment of *case 2*, as well (Fig. 2).

### Preceptor vs. non-preceptor real knowledge and self-assessment

There was no statistically significant difference related to their precepting teaching experience between the two groups of pharmacists (preceptors and non-preceptors) in their knowledge scores (case 1 and case 2). More than half of the preceptors had unsatisfactory or not good enough knowledge of case 1 and 38.8 % of case 2, while 48.4 % of the non-preceptors had unsatisfactory or not good enough knowledge of both cases (Table 4).

**Table 4:** Preceptor vs. non-preceptor real score knowledge and self-assessment of knowledge

	Preceptorship experience		*p* value^a^
	no	yes	
Case 1			
unsatisfactory	5 (16.1 %)	14 (28.6 %)	0.413
not good enough	10 (32.3 %)	12 (24.5 %)	
average	5 (16.1 %)	9 (18.4 %)	
good	6 (19.4 %)	6 (12.2 %)	
very good	5 (16.1 %)	8 (16.3 %)	
Case 2			
unsatisfactory	13 (41.9 %)	14 (28.6 %)	0.370
not good enough	2 (6.5 %)	5 (10.2 %)	
average	0 (0 %)	1 (2 %)	
good	1 (3.2 %)	2 (4.1 %)	
very good	15 (48.4 %)	27 (55.1 %)	
Self-assessment of Case 1			
unsatisfactory	0 (0 %)	0 (0 %)	0.099
not good enough	0 (0 %)	0 (0 %)	
average	13 (41.9 %)	11 (22.9 %)	
good	16 (51.6 %)	33 (68.8 %)	
very good	2 (6.5 %)	4 (8.3 %)	
Self-assessment of Case 2			
unsatisfactory	0 (0 %)	0 (0 %)	0.041
not good enough	0 (0 %)	0 (0 %)	
average	11 (35.5 %)	7 (14.6 %)	
good	17 (54.8 %)	33 (68.8 %)	
very good	3 (9.7 %)	8 (16.7 %)	

^a^Mann-Whitney U test

Nevertheless, there was a statistically significant difference in the self-assessment of knowledge between preceptors and non-preceptors. In case 1, 77.1 % of preceptors self-assessed themselves as good or very good, while 58.1 % of the non-preceptors self-assessed themselves as good or very good. The self-assessment of case 2 had higher scores in both groups (Table 4).

## Discussion

We believe this is the first study conducted in the Balkans and potentially wider combining external observation with pharmacists’ self-assessment of their knowledge as well as investigating the significance of preceptorship experiences. Overall, we believe based on our findings that knowledge-based assessments, including objective structured examinations similar to the one undertaken in this study, are particularly useful to help accurately assess pharmacists’ competencies in certain fields. Evaluation of knowledge by examination (multiple-choice) is a known method for assessment of knowledge [53]. Having said this, we believe it is difficult to comprehensively test clinical knowledge only with paper-based examinations. Consequently, we consider objective (external) knowledge-based assessments as a sustainable tool for the future. In our study, we used a two assessment tools created by NGT: objective external assessment by two case scenarios and self-assessment in order to get better insight (considering the possible disconnect between knowledge and practice behavior) into actual practice behavior.

Of concern is that this study showed that pharmacists surveyed possessed insufficient clinical knowledge about the safe use of contraceptives. This agrees with the findings of Koračević et al. who believed there is insufficient recognition of drug-related problems among community pharmacists in the Balkan countries which may be due to a lack of clinical issues explored during their education. Consequently, there is a need for a continuous improvement of knowledge and skills post-qualification, which is essential for a proactively patient-focused approach [4]. This is also reflected in another study conducted in Serbia by Stojkov et al. where there were concerns with low scores of pharmacists’ competencies in diagnosis (minor aliments) and patient counseling [16]. Half of the surveyed pharmacists in a Serbian study had also poor knowledge of emergency contraception [38]. Another concern is that clinical knowledge in this thematic issue did not increase with pharmacists’ experience as they progress through preceptors’ experience unlike a recent study from Croatia which did show an increase in pharmaceutical care competency over time [54] and physicians increasing their competency as they progress to senior registrars [33]. However, most of the pharmacists in our study were 25 to 35 years old. In B & H, continuing education (CE) is necessary for the professional license renewal with a defined sum of credits necessary over a five year cycle through CE activities for continued practicing. Through these activities, community pharmacists are educated about different thematic issues. Our findings and other publications suggest B & H professional organizations need to provide a comprehensive reproductive health course on contraception, emergency contraception and medical abortion both online or in person through CE programs to appreciably improve the knowledge of pharmacists in this area [36, 37]. Pharmacy personnel need to be provided with clear information and guidelines for provision of SRH approaches especially as evidence suggests that pharmacies have qualities which make them convenient points of SRH commodity access [40].

Our finding also demonstrates pronounced under prescribing of contraceptives in B & H with nearly 60 % of pharmacists only dispensing between two and ten oral contraceptives during a month. These study findings also highlight an underlying lack of support for SRH activities amongst policymakers, medical and pharmacy communities in B & H. This issue needs to be promptly addressed to reduce the frequency of medical abortions. This is similar to the situation in Serbia where the study of Milosavljević showed that half of the surveyed gynecologists had moral/ethical objections to certain contraceptive methods, and would not offer them to patients [38].

Another concern is that the majority of pharmacists (85 %) in our survey would decline ECP provision because the female patient was a minor (under 18 years old). Gonsalves et al. in their systematic review identified these reservations of pharmacists as centered around a general concern that SRH approaches (mostly ECPs) were not safe for the young or that the young would not be able to take them as directed [40]. Another reason for pharmacists’ reservation is that increased availability of SRH commodities (ECPs, in particular) could result in “risky and promiscuous” behavior among the young [40]. This is again a concern given the high rate of abortions in B & H and the high rate globally of unsafe abortions [35, 36]. ECPs are available directly from pharmacies in B & H; consequently, it is of considerable importance for the future to consider training pharmacists and developing streamlined protocols that do not deter women, including minors, from obtaining ECP so that the use of ulipristal acetate can be encouraged whenever appropriate. Strong support by professional regulatory agencies can enable the full potential of ulipristal acetate to reduce unwanted pregnancies to be achieved [55]. The development of ECP guidelines and protocols in B & H can capitalize on the those developed by other countries around the world. In addition, developing guidelines and protocols for ECP builds on the situation in other countries and for other conditions in B & H [12, 56], and potentially including these into the professional standards of B & H and other West Balkan countries. Until the guidelines and protocols are built, pharmacists should be guided by the Summary of medical product characteristics (SmPC), Register of medicines and internal procedures of each pharmacy (if such exists). This means that every woman with a menstrual cycle should be allowed to use ulipristal acetate, including adolescents. Ulipristal acetate is currently registered in B & H as a non-prescription medicine, while levonorgestrel is registered as a prescription-only medicine.

The study also demonstrated that pharmacists lack insight into their own strengths and weaknesses and tend to overestimate their abilities. Other studies have also confirmed these phenomena and effects on pharmacists and pharmacy students as well as showing that those with the highest scores tended to underestimate themselves (Fig. 2) [53, 57]. Similarly, as mentioned, physicians also do not always appear to accurately self-assess their performance [30].

One study also showed that pharmacy preceptors overestimated their skills related to teaching compared to students’ perception of their real performance [58]. This is important as it has considerable implications for improving pharmaceutical care for patients in the future. Overall, more emphasis should be placed on knowledge-based assessments as objective (external) examinations rather than on pharmacists’ perceptions to evaluate lifelong learning outcomes. This is because there is a need for continuous improvement of knowledge and skills for patient-focused approaches in pharmacy practice [4, 12, 30]. Implementation of clinical pharmacy skills in pharmacy practice improves therapeutic and financial outcomes to the benefit of all key stakeholders [18, 19, 59], and we will be looking to develop these to improve the availability of contraception in B & H in the future to reduce the number of unwanted pregnancies and abortions and more generally for patients.

Our study identified the lack of information about B & H pharmacists’ and physicians knowledge and attitudes toward woman reproductive health, such have been analyzed in Serbia [38]. Our results revealed the lack of pharmacists’ knowledge and attitudes in recognizing the symptoms of possible thrombosis in female patient which is a concern. This emphasizes the need for CE activities, which are needed more than ever during the pandemics given the level of misinformation and the recognized role of pharmacists in providing guidance on possible prevention and treatment approaches [1–3, 14, 15]. This also includes the possibility of SARS-CoV-2 infections aggravating the risk of thromboembolic events associated with combined contraceptives and other estrogen therapies [60–62]. Overall, we believe our findings and implications will be of interest to other countries seeking to reduce the extent of unwanted pregnancies and unsafe abortions.

We are aware that our study has a number of strengths and weaknesses. Strengths include highlighting the importance of enhancing the future education of pharmacists in the area of SRH as well as proposing a novel objective and easy-to-implement assessment tool. This is because we believe assesment methods and tools used in our study have not been fully explored in scientific literature among pharmacists and other health care professionals. Alongside this, we could recognize the capacity for the further development of these tools for other health issues and upgrading for the purpose of measuring pharmacists’ performance in community pharmacies. The use of NGT in order to develop our cases we believe is also an additional strength of our study. The limitations include that fact that our questionnaire was not statistically validated and the sample of pharmacists was mostly young health professionals aged between 25 and 35 years old.

## Conclusions

Pharmacists’ clinical knowledge regarding the safe use of oral and emergency contraceptives was shown to be lacking among community pharmacists in B & H. This needs to be addressed given the high rate of abortions in the Balkans and low and inconsistent activities regarding SRH (for all health professionals), which is jeopardizing the health and well-being of the woman and families in our region. We anticipate that the clinical knowledge of pharmacists in this area will increase with new graduate curricula which have clinical pharmacy and pharmacy practice courses, and part of these courses will be dedicated to this thematic issue. Future studies should assess the impact of these planned educational activities on the new pharmacy curricula for undergraduate students and CE for practicing pharmacists on professional development of pharmacists regarding the safe use of oral and emergency contraceptives in B & H to provide guidance to other similar countries. This is because community pharmacists have considerable potential to be a source of SRH approaches but only with adequate training and professional development. The self-enhancement or self-evaluation bias, i.e. the lack of insight of pharmacists into their own weaknesses regarding their knowledge, also needs to be investigated further as part of the professional practice of pharmaceutical care and wider, and we will be following this up in future research projects.

All authors read and approved the final manuscript. All authors agreed to be personally accountable for the author’s own contributions and ensured that questions related to the accuracy or integrity of any part of the work, even ones in which the author was not personally involved, are appropriately investigated, resolved, and the resolution documented in the literature.

## Supplementary Information

**Additional file 1.**

**Additional file 2.**

## Data Availability

Not applicable.

## Abbreviations

LMICs: Low- and middle-income countries.
B & H: Bosnia and Herzegovina.
SRH: Sexual and reproductive health.
NGT: The nominal group technique.
ECP: Emergency contraceptive pill.
CE: Continuing Education.
IRB: Institutional Review Board.
COC: Combined oral contraceptive.
