# Supplementary material for: Pharmacists’ clinical knowledge and practice in the safe use of contraceptives: real knowledge vs. self-perception and the implications
Source: BMC Med Educ. 2021 Aug 16;21:430. doi: 10.1186/s12909-021-02864-9 (PMC8365278; doi:10.1186/s12909-021-02864-9)
Supplement: Supplementary file 1 — Additional file 1. [file 12909_2021_2864_MOESM1_ESM.docx]

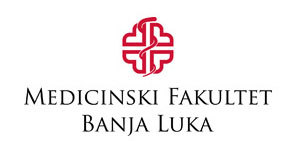


FACULTY OF MEDICINE

BANJA LUKA

**Cross-sectional study of knowledge and attitudes of pharmacists in Bosnia and Herzegovina regarding the use of oral and emergency contraception**

*Dear colleagues,*

*Please fill in the following questionnaire. The questionnaire consists of three domains. The first domain consists of two* ***case studies****, which scenarios were invented for the purposes of this research. In the second domain of the questionnaire, you should do a self-evaluation (to rate on a scale of five claim how you think you advised the patient in the first part of the questionnaire) and provide information about your dispensing practice. The third domain of the questionnaire serves to collect your personal information.*

*Your participation in the research is voluntary and anonymous.*

**I – Potential cases for practice**

(*based on 2 given cases, please circle one answer within each claim; in the last line of questioner in both cases, you can write down an advice that is not offered as claim and that you would give to patient*)

CASE 1

A permanent female patient (26 years old) comes to the pharmacy and turns to a pharmacist for advice, because she is going to America (by airplane) with her boyfriend in two days. She has been planning this trip for a long time, and this morning she noticed that her lower leg was swollen and she was in pain. She wants pharmacist to help her, because she knows that she will walk a lot during the trip, because they are travelling as tourists. She suggests that whatever we recommend to her should last for 10 days, because she is not sure whether she will be able to get the same medicines without a prescription in pharmacies in America. The patient wants to take something stronger for the headache, because paracetamol does not always help her.

After gathering information from the patient, a pharmacist finds out that the patient is a smoker, has no illness, denies leg trauma, that she took 500 mg of paracetamol this morning for a severe headache, and otherwise only takes oral contraceptives.

1. We suggest to a patient a gel with 30 000 i.j. heparin, to reduce swelling. Apply gently twice a day, from the foot towards the knee.
2. **Yes**
3. No
4. We suggest to a patient a diclofenac against pain, to be massaged twice a day on the painful and swollen area.
5. Yes
6. **No**
7. We suggest to a patient a heparin gel to reduce swelling and diclofenac gel against pain. Massage alternately.
8. Yes
9. **No**
10. We suggest to a patient a therapy for headache, in addition to paracetamol, a dexketoprofen:
11. Yes
12. **No**
13. Acetylsalicylic acid is contraindicated:
14. Yes
15. **No**
16. We suggest to a patient to walk and avoid prolonged sitting position:
17. **Yes**
18. No
19. We advise to a patient:
20. To continue to take oral contraceptives
21. **To stop taking oral contraceptives, and in the following period to use other methods of protection (e.g., condoms)**
22. We advise to a patient:
23. That there is no need to contact a doctor
24. To contact a doctor if the recommended therapy does not help.
25. **To contact a doctor as soon as possible, obligatory before departure**.

Give the reason for sending/not sending the patient to a doctor:

__________________________________________________________

1. We have patient’ contact phone in the computer, and we will contact her during today or tomorrow.
2. **Yes**
3. No
4. Here you can write an additional advice for the patient if you want:

________________________________________________________________________________________________________________________________________________________________________________________________________________________

CASE 2

A female patient who appears worried and scared comes to the pharmacy. In a conversation with the patient, the pharmacist finds out that she is 16 years old, that she had an unprotected intercourse with her boyfriend 4 nights ago, and that she is afraid that she could have become pregnant that very evening. The patient is a smoker, denies illness, has had regular cycles and regular sexual intercourse from last year. Normally, they use the method of "interrupted intercourse" for protection, because the boyfriend does not want to use a condom. She did not visit a gynecologist until now, for fear that her parents would find out because she lives in a small community. She says that she has read on the internet about pills called the “Morning-after”, but that she knows nothing about them. She states that she cannot talk to her mother about this, because she is afraid of her reaction. She grew up in a family with strong religious beliefs and it is not allowed to have an abortion, so she also has doubts about taking the pill she read about on the Internet. At the same moment, she starts crying, because she thinks that she is too young for a child and that she is not ready for a family. Even in case the pill causes an abortion (and her parents find out) her life will be ruined.

1. I have a conversation with the patient in the part of the pharmacy for confidential conversations.
2. **Yes**
3. No
4. I try to finish the counseling as soon as possible because I notice that the patient is very uncomfortable because of other patients in the pharmacy.
5. Yes
6. **No**
7. In this case the medicine to choose is:
8. levonorgestrel
9. **ulipristal - acetate**
10. none of the above
11. Given that the patient is a minor (under 18 years old), I do not issue emergency contraception, but I refer her to see a doctor:

a) Yes

b) **No**

1. Since the patient is a smoker, and smoking is a contraindication for the use of emergency contraception, I do not give the patient pills for the “Morning-after”.
2. Yes
3. **No**
4. I explain to the patient that: the “Morning-after” pills work by inhibiting ovulation, thus not terminating but preventing pregnancy.
5. **Yes**
6. No
7. I explain to the patient that the “Morning-after” pills terminate the pregnancy in over 85% of cases.
8. Yes
9. **No**
10. I advise the patient that after taking the “Morning-after” pill, in case of delay of the next cycle for more than 10 days: she should take a pregnancy test and consult a gynecologist.
11. **Yes**
12. No
13. I advise that, since the patient has regular intercourse with her boyfriend, she should still schedule an appointment with a gynecologist and consider starting to use oral contraceptives as a method of effective protection against unwanted pregnancy.
14. **Yes**
15. No
16. I advise that the method of protection she uses with her boyfriend (interrupted intercourse) is the recommended method of protection against unwanted pregnancy, because in this way she does not take oral contraceptives that carry a high risk of side effects.
17. Yes
18. **No**
19. Here you can write an additional advise for the patient if you wish:

________________________________________________________________________________________________________________________________________________________________________________________________________________________

**II – Self-evaluation of knowledge and dispensing practice**

1. Please do a self-evaluation now, i.e. circle on a given scale how you feel you have advised the patient in case 1:

Very good Good Average Not good enough Bad

1. Please do a self-evaluation now, i.e., circle on a given scale how you think you have advised the patient in case 2:

Very good Good Average Not good enough Bad

1. How many boxes of oral contraceptive pills you issue on average in one month:
2. One or none
3. 2-10
4. More than 10
5. How many boxes of emergency contraceptive pills you issue on average in one month:
6. One or none
7. 2-10
8. More than 10

**III – Demographic and professional work-related information**

1. Your gender:
2. Female
3. Male
4. Your age:
5. <25
6. 25-35
7. 36-45
8. 46-55
9. >56
10. You are:
11. Pharmacist
12. Other ____________
13. You work in:
14. Public (community) pharmacy
15. Hospital pharmacy
16. Elsewhere __________________
17. How many years of work experience do you have in pharmacy:
18. 1-5 years
19. 6-10 years
20. 11-20 years
21. > 20 years
22. List previous work experience in relation to the current institution:

*(you can circle more than one answer)*

1. Wholesale pharmacy
2. Marketing Associate
3. Production of medicines
4. Other
5. I have no previous experience
6. Do you have experience in a managerial position or are you currently in that position:
7. Yes
8. No
9. Have you been a mentor/preceptor to students/interns during your internship?
10. Yes
11. No
12. If you were a mentor/preceptor, how many years of experience do you have in mentoring:
13. <5
14. 6-10
15. 11-20
16. >20
17. Where do you see yourself in 5 years:
18. in pharmacy, in the same institution
19. in pharmacy, but in another institution
20. I do not see myself in pharmacy
